# Metabolic adaptations in prostate cancer

**DOI:** 10.1038/s41416-024-02762-z

**Published:** 2024-07-05

**Authors:** Mikel Pujana-Vaquerizo, Laura Bozal-Basterra, Arkaitz Carracedo

**Affiliations:** 1 Center for Cooperative Research in Biosciences (CIC bioGUNE), Basque Research and Technology Alliance (BRTA), Bizkaia Technology Park, Building 801A, 48160 Derio, Spain; 2 Centro de Investigación Biomédica En Red de Cáncer (CIBERONC), 28029 Madrid, Spain; 3 Traslational Prostate Cancer Research Lab, CIC bioGUNE-Basurto, Biobizkaia Health Research Institute, Baracaldo, Spain; 4 Ikerbasque, Basque Foundation for Science, Bilbao, Spain; 5 Biochemistry and Molecular Biology Department, University of the Basque Country (UPV/EHU), Leioa, Spain

**Keywords:** Cancer metabolism, Urological cancer

## Abstract

Prostate cancer is one of the most commonly diagnosed cancers in men and is a major cause of cancer-related deaths worldwide. Among the molecular processes that contribute to this disease, the weight of metabolism has been placed under the limelight in recent years. Tumours exhibit metabolic adaptations to comply with their biosynthetic needs. However, metabolites also play an important role in supporting cell survival in challenging environments or remodelling the tumour microenvironment, thus being recognized as a hallmark in cancer. Prostate cancer is uniquely driven by androgen receptor signalling, and this knowledge has also influenced the paths of cancer metabolism research. This review provides a comprehensive perspective on the metabolic adaptations that support prostate cancer progression beyond androgen signalling, with a particular focus on tumour cell intrinsic and extrinsic pathways.

## Introduction

The androgen receptor (AR) is a central player in the biology of the prostate, operating as a nuclear receptor essential for normal prostate development and function [1]. AR mediates the effects of androgens and regulates the expression of genes involved in prostate growth, maintenance, and differentiation. Beyond developmental stages, AR also influences prostate health throughout adulthood [2]. AR signalling is linked to the onset and progression of prostate cancer (PCa), where it becomes a primary driver of tumour growth. Therefore, inhibition of AR function represents the targeted therapy in this disease [3]. AR reprograms PCa cellular metabolism, creating a unique molecular scenario that has been documented for the last 100 years [4]. Nevertheless, the complexity underlying cellular metabolism extends beyond AR signalling, which is envisioned to offer innovative therapeutic opportunities. Our current understanding of cellular metabolism encompasses aspects such as the tumour microenvironment (TME) or diet. In this review, we will explore major metabolic pathways supporting PCa progression and metastasis, with special emphasis on tumour cell-intrinsic and extrinsic glucose, lipid and one-carbon metabolism (1 C metabolism), while other relevant processes including the connection between metabolism and epigenetics will be left out of the scope of this work. Furthermore, we will incorporate new evidence from other tumour types to identify shared characteristics that can apply to PCa.

## Major metabolic alterations in prostate cancer cells

### Glucose metabolism

#### Glycolysis and the Warburg effect

**Fig. 1:**
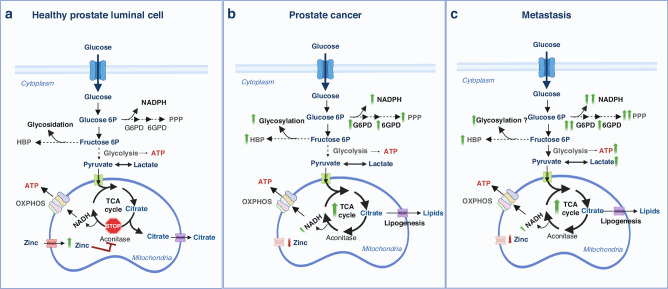
Schematic overview of the metabolic rewiring occurring in prostate epithelial cells during the different stages of cancer progression. **a** Healthy prostate luminal cells accumulate high levels of zinc (due to the overexpression of its transporter), leading to the inhibition of mitochondrial aconitase, the key enzyme responsible for the citrate-isocitrate conversion in the TCA cycle. This inhibition results in the truncation of the TCA cycle and citrate accumulation and secretion. As a result, normal prostate epithelial cells are characterized by an inefficient OXPHOS. **b** In prostate cancer cells, intracellular zinc levels are significantly reduced (due to a decreased expression of its transporter); this leads to the reactivation of aconitase, restoring the citrate-isocitrate conversion, and consequently of the TCA cycle and OXPHOS metabolic pathways. In addition, both the hexosamine biosynthesis pathway (HBP) resulting in glycosylation and the pentose phosphate pathway (PPP) that generates NADPH and nucleotides are upregulated in PCa cells. **c** Metastatic PCa cells exhibit the Warburg effect with persistent TCA cycle/OXPHOS and PPP activity. Created with BioRender.com.

Glycolysis metabolises glucose to pyruvate via a series of intermediate reactions, generating ATP and NADH (Fig. 1). Cancer cells often exhibit increased glycolytic activity to generate lactate, even in the presence of oxygen, known as aerobic glycolysis or the Warburg effect [5]. Despite its lower efficiency compared to oxidative phosphorylation (OXPHOS) in the mitochondria, cancer cells heavily depend on this pathway to produce energy. It is important to state that the increase of anaerobic glucose utilisation does not imply a reduction in mitochondrial OXPHOS activity, in contrast to the initial hypothesis of Dr. Otto Warburg [6]. Although several hypotheses have been proposed, the reason why proliferating cells metabolise glucose predominantly to produce lactate remains elusive. Two complementary publications argue that when the demand for NAD+ exceeds the demand for ATP, resulting in the saturation of the mitochondrial NADH, tumour cells enforce aerobic glycolysis even in the presence of oxygen [7, 8]. These metabolic adaptations have been brought from bench to bedside through different approaches. On the one hand, aerobic glycolysis results in an elevated demand for glucose, which has inspired the development of cancer-monitoring strategies based on the uptake of ^18^F-fluorodeoxyglucose with positron emission tomography (PET) [9]. On the other hand, although alterations in copy number have been reported in glycolysis-promoting genes [10], deregulated mRNA expression represents a key contributing factor for aerobic glycolysis, which supported the development of transcriptomic gene signatures in different cancer types [11–13].

Androgen receptor plays a predominant role in controlling the expression of growth-promoting and anti-apoptotic genes involved in various metabolic processes, such as glycolysis [14]. Glucose transporters GLUT1 and GLUT2 are regulated at the transcriptional level by the AR [15–17], whereas control by AR-independent factors such as SOX2 and MYC promotes prostate cancer progression, lineage plasticity, and therapy resistance [18, 19]. Interestingly, the Warburg effect is observed both in localised PCa [20, 21], as well as in advanced disease or metastatic lesions [22–24]. In this line, it has been reported that the highest lactate levels are found in patients with *PTEN* loss, a genetic feature of advanced PCa [25]. The activation of the PI3K–AKT–mTOR signalling pathway is believed to be a key factor in PTEN-deficiency-driven prostate tumorigenesis promoting aerobic glycolysis [26, 27]. Inhibition of MCT4 (a plasma membrane lactic acid transporter), has been postulated as a therapeutic strategy to reduce glycolysis and lactic acid secretion in neuroendocrine prostate cancer (NEPC), a subtype of aggressive PCa [28]. In NEPC cell lines, inhibiting MCT4 expression reduced cell proliferation in vitro and glucose metabolism by downregulating glycolytic genes. However, data about the effectiveness and toxicity of MCT4 inhibition in NEPC models in vivo are still lacking.

Despite the relevance of glycolysis for tumour cells, accumulating evidence sustains that both the tricarboxylic acid (TCA) cycle and the OXPHOS pathway are still present and active in the metastatic setting [6, 29–36], which could complicate the use of lactate-targeted therapies [27]. A promising new small molecule, BKIDC-1553 (which exhibits good safety and pharmacologic properties), has been shown to selectively inhibit the growth of PCa cell lines through its anti-glycolytic activity. This is achieved by inhibiting hexokinase 2, as reported in a preclinical xenograft model of advanced PCa. The selective growth inhibition activity of BKIDC-1553 is equivalent to that of enzalutamide [37]. All these results demonstrate the complexity and impact of metabolic interactions within tumours and in different stages, highlighting the importance of studying drug effects in diverse metabolic scenarios.

#### Tricarboxylic acid cycle (TCA) and oxidative phosphorylation (OXPHOS)

The TCA comprises a series of mitochondrial chemical reactions and is responsible for producing energy and metabolic intermediates. It begins with the conversion of acetyl-CoA (which is produced from the breakdown of carbohydrates, fats, and proteins) and oxaloacetate into citrate. Through a series of enzymatic reactions, citrate is transformed, resulting in the production of carbon dioxide and molecules carrying high-energy electrons, namely NADH and FADH2. These electron carriers are subsequently utilised for OXPHOS, a process that occurs in the inner mitochondrial membrane. OXPHOS involves the electron transport chain (ETC) and a proton gradient, ultimately leading to the production of ATP (Fig. 1). Despite the initial perception of a general reduction of the use of glucose derivatives in the mitochondria, recent evidence shows that there is tumour-type specificity in this reprogramming. Whereas pancreas, lung and colon tumours exhibit a slower ATP production than healthy tissues, breast cancer-derived metastases reportedly show faster TCA cycle rate than orthotopic primary tumours [38]. In line with these results, an increase in OXPHOS gene expression was detected in melanoma brain metastasis (MBM) by direct metabolite profiling and [U-^13^C]-glucose tracing in vivo [39], which is associated to increased sensitivity of these lesions to pharmacological OXPHOS inhibition [39]. However, later clinical trials to analyse the therapeutical potential of the same OXPHOS inhibitor in advanced solid tumours and acute myeloid leukaemia showed only modest target inhibition and limited antitumour activity at tolerated doses and led to discontinuation of the trials due to neurotoxicity [40]. Interestingly, tumour cells exhibit changes in the TCA that go beyond the regulation of its activity. Mutations in the TCA cycle or the ETC machinery induce alternative metabolic routes, such as reductive carboxylation observed in various cancer types [41–47]. Beyond the effect of mutations altering the TCA cycle, specific cell state transitions are accompanied by profound TCA reprogramming comprising the extramitochondrial use of citrate that regenerates oxaloacetate [48].

Prostate epithelial cells do not oxidise the produced citrate like most normal cells due to truncated TCA cycle [49–53]. Instead, luminal prostate cells, but not basal cells [54], accumulate high mitochondrial zinc (Zn^2+^). Zn^2+^ inhibits the mitochondrial enzyme (m)-aconitase, responsible for citrate oxidation, and the accumulated citrate is subsequently secreted into the prostatic fluid. While basal cells preferentially generate citrate through pyruvate dehydrogenase, luminal cells predominantly generate citrate through pyruvate carboxylase activity [54]. Metabolism of pyruvate, aspartate, glutamine and branched-chain amino acids (BCAA) might contribute to replenishing metabolites for the truncated TCA cycle in PCa [55, 56]. AR induces a metabolic reprogramming encompassing hZIP1 zinc transporter downregulation that leads to low mitochondrial zinc levels [57, 58] and m‐aconitase reactivation, restoring the TCA cycle [59], and increasing the susceptibility of PCa cells to OXPHOS inhibitors [60].

Oxidative phosphorylation can be targeted by restricting the supply of NADH or by directly inhibiting components of the ETC. Mutations in the mitochondrial DNA encoding for OXPHOS machinery promote Warburg-like metabolism and an anti-tumour immune response [61]. Targeting the TCA cycle by compromising mitochondrial substrate trafficking might also be an effective strategy. For example, metformin and rotenone, inhibitors of complex-I (CI) of the ETC, inhibit proliferation in several human cancer cell lines, including PCa [62–64]. Evidence has shown that metformin has multiple antineoplastic effects through AMPK-dependent and independent mechanisms, namely the alteration of IGF-1 signalling pathways, suppression of AR or mTOR pathway, and lipogenesis. In line with this notion, there is evidence for reduced mortality in PCa patients treated with metformin [64]. The rotenone derivative deguelin exhibits antitumoural activity in preclinical mouse models of PCa based on the combined loss of *Pten* and *Trp53* [65]. This effect is associated to the alternative use of the ETC by *Pten*-deficient cells, which consume ATP through mitochondrial complex V instead of producing it. This observation could be translated to the use of CI inhibitors in PCa patients stratified by *PTEN* status. Whereas most PCa research is focused on the effect of AR signalling promoting TCA cycle [66, 67], a deeper understanding of the AR-independent metabolic alterations is lacking and could be critical when designing therapeutic strategies in castration-resistant patients.

#### Amino acid metabolism or the pentose phosphate pathway (PPP)

The PPP is a main producer of NADPH and nucleic acid precursors [68], which helps tumour cells balance the redox status. Tumour cells exhibit deregulation of oncogenes and tumour suppressor genes that control this pathway [69]. Genetic deficiency in glucose-6-phosphate dehydrogenase (G6PD), one of the rate-limiting enzymes of the PPP, is a common inherited enzyme defect and occurs almost exclusively in males [70–72]. There is increasing evidence that this deficiency may offer protection against stomach, colon, and liver cancer. Conversely, G6PD upregulation has been associated with higher cancer risk [73]. In fast proliferating cells, a high NADP^+^/NADPH ratio activates G6PD to support NADPH production, leading to reductive biosynthesis of fatty acids and nucleotides. Furthermore, NADPH promotes cell survival under oxidative stress conditions such as mitochondrial dysfunction [74]. Upregulated G6PD activity is observed in various cancers, including papillary thyroid carcinoma, colorectal, renal, hepatocellular, breast, and PCa [75–81]. Mechanistic research in PCa cell lines suggests that AR-mediated regulation of the PPP occurs through upregulation of G6PD in response to mTOR complex 1 activation, leading to the production of nucleotide precursors for DNA synthesis and NADPH to promote lipogenesis [82] (Fig. 1). Indeed, PPP and G6PD have been proposed as metabolic targets for PCa bone metastasis treatment [83]. In vitro, genetic and pharmacological G6PD inhibition decreased cancer growth and migration, leading to alterations in cellular redox balance and heightened sensitivity to chemotherapy. In vivo, G6PD genetic ablation resulted in the reduction of bone metastatic burden. A recent study revealed that another PPP-related enzyme, 6PGD, plays a key role in PCa growth and survival by counteracting oxidative stress and uncovered a novel feedback mechanism linking 6PGD and the AR signalling axis that opens a new therapeutical window of co-targeting AR and the PPP [84]. Genetic or pharmacological inhibition of 6PGD using physcion and S3 showed anticancer activity in aggressive, castration-resistant disease models as well as patient-derived tumour explants, partly due to increased oxidative stress. Targeting of 6PGD was associated with two important tumour-suppressive mechanisms: firstly, it increased the activity of the AMP-activated protein kinase (AMPK); secondly, it enhanced AR ubiquitylation, leading to a reduction in AR protein levels and activity. Pharmacological co-targeting of both factors was more effective in suppressing the growth of PCa cells than single-agent therapies, indicating positive feedback between AR and 6PGD. All these findings suggest that the PPP could be a valuable source of targets for anticancer drug design and therapeutic combination.

#### Hexosamine biosynthetic pathway (HBP)

The HBP is a metabolic route that redirects 2–5% of glucose-derived carbons away from glycolysis in non-cancer cells. It comprises the conversion of the glycolytic intermediate fructose-6-phosphate to produce UDP-N-acetylglucosamine (UDP-GlcNAc) [85]. UDP-GlcNAc serves as a substrate for various cellular processes, including protein glycosylation—a crucial post-translational modification where sugars are attached to proteins and lipids. Cancer cells upregulate the flux towards the HBP and UDP-GlcNAc synthesis by increasing glucose and glutamine intake or in response to oncogenic-associated signals like Ras [86], mammalian target of rapamycin complex 2 (mTORC2) [87, 88], and transforming growth factor beta (TGF-β) [89]. In line with increased UDP-GlcNAc levels, breast [90, 91], lung [92], colon [92], liver [93], endometrial [94], cervical [95], pancreatic cancer [96] and PCa [97] cells exhibit increased O-GlcNAcylation (Fig. 1). There are diverse molecular alterations that converge on increased synthesis of glycans. The second-rate limiting enzyme of the HBP, UAP1 [98–100], is elevated in PCa, which protects tumour cells from ER stress-induced cell death, thus postulating it as a viable target for cancer therapy. An enzyme involved in the conjugations process, the glycosyltransferase GALNT7, is also upregulated in PCa tissues and promotes prostate tumour growth [101]. Lessons from other tumour types reveal the metabolic crosstalk that balances the use of glucose intermediary metabolites. Loss of the PHGDH, an enzyme involved in glucose-derived serine biosynthesis promotes metastasis by rewiring glucose towards HBP, thus increasing integrin glycosylation [102].

Increased glycosylation influences the structural diversity in proteins, including sialylation, fucosylation, *O*-β-*N*-acetylglucosylation, and the presence of cryptic and high-mannose *N*-glycans and proteoglycan alterations [103]. Based on the evidence presented, therapeutic targeting of HBP rises as an innovative strategy to selectively affect cancer cells, as non-transformed cells would be more resilient to the perturbation in O-GlcNAcylation [104, 105]. Hexosamine analogues could serve this purpose since they exhibit antitumoral properties. Other promising therapeutic strategies in preclinical models involve the pharmacological inhibition of OGT, the HBP enzyme that catalyses the addition of the GlcNAc residue to target proteins. On the one hand, inhibiting O-GlcNAcylation in PCa cells reduced the expression of matrix metalloproteinase MMP-2, MMP-9, and VEGF, resulting in inhibition of invasion and angiogenesis mediated by the regulation of the oncogenic transcription factor FoxM1 [104]. On the other hand, OGT inhibition reduced the proliferation of PCa cells due to sustained loss of c-MYC [97]. As a note of caution, the requirement of HBP for PCa cells might be tumor stage-dependent. In fact, castration-resistant PCa shows decreased HBP metabolite and enzyme levels, suggesting that targeting the pathway in this pathological setting could have unpredictable biological consequences [106]. Overall, understanding the function and composition of glycoproteins and glycans across all stages of PCa will likely be crucial to improving disease management. The relevance of the cell surface glycan profile for cell-cell interactions anticipates that HBP and glycosylation rewiring will have profound implications in the interactions of tumour cells with the TME.

### Lipid metabolism

Lipid metabolic reprogramming encompasses alterations in various aspects of lipid metabolism, including synthesis, storage, and catabolism [107]. One significant adaptation is the upregulation of lipogenic pathways, where cancer cells enhance the production of fatty acids and other lipid components to sustain their rapid growth. This increase in lipogenesis often involves the activation of key enzymes such as ATP citrate lyase (ACLY) and acetyl-CoA carboxylase (ACC), driven by oncogenic signalling pathways like the PI3K/Akt/mTOR axis and MYC [108]. Additionally, cancer cells exhibit changes in lipid uptake and utilization, relying on both endogenous and exogenous lipid sources to sustain their metabolic needs.

**Fig. 2:**
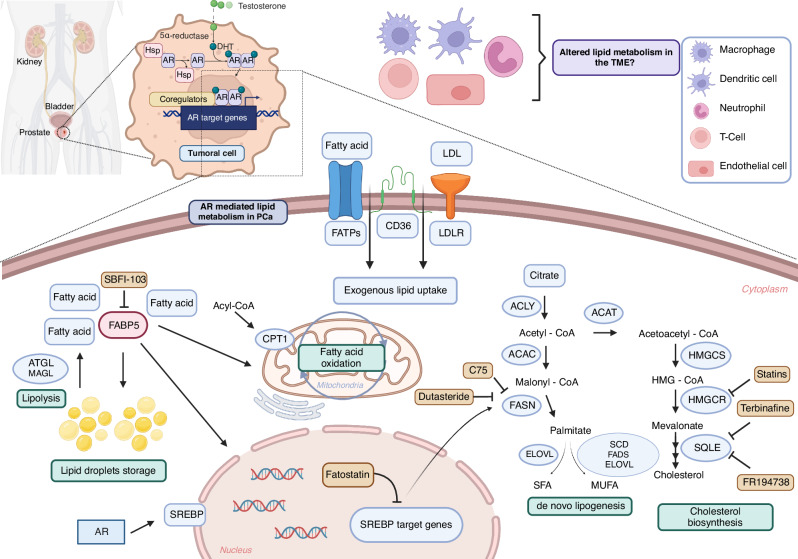
The landscape of lipid metabolism in PCa. The figure illustrates the key aspects of lipid metabolism in PCa, mainly driven by AR-mediated cellular reprogramming of tumoral cells. In response to AR signalling, PCa cells exhibit an augmented de novo lipogenesis through transcriptional regulation by SREBPs. Mitochondrial fatty acid oxidation, via upregulation of the CPT1 transporter, generates energy for proliferation. In addition, PCa cells increase exogenous fatty acid uptake through upregulated CD36 and FATPs. FABPs play a role in the intracellular compartmentalization of fatty acids. Dysregulation of cholesterol metabolism also represents a characteristic feature of prostate tumours. The main enzymes and regulators taking part in these pathways are highlighted, alongside various inhibitors studied for potential therapeutic interventions. Finally, the study of lipid metabolism in cells comprising the TME emerges as an important strategy for future research. Abbreviations: AR; androgen receptor, DHT; dihydrotestosterone. Created with BioRender.com.

Dysregulation of lipid metabolism is considered a hallmark in PCa [109]. These tumour cells display distinct alterations in lipid metabolism compared to normal prostate counterparts, and these changes are associated with tumour growth, survival, and metastasis [109]. Whereas alterations in oncogenes and tumour suppressor genes (*p53* loss, *PTEN* loss, *PI3K* mutations) that are shared across different tumour types can alter this process, enhanced lipid metabolism in PCa is predominantly driven by AR signalling [110, 111]. Indeed, AR controls the transcription of enzymes involved in fatty acid synthesis and oxidation to fulfil the bioenergetic and anabolic demands of PCa cells, and it also regulates lipid uptake and storage, cholesterol, and phospholipid metabolisms [112] (Fig. 2).

#### De novo lipogenesis (DNL)

PCa is characterised by augmented DNL in both in early and late stages of the disease [113]. This pathway is tightly regulated and produces fatty acids from non-lipidic precursors. The primary substrate in fatty acid synthesis is acetyl-CoA, which is carboxylated by acetyl-CoA carboxylase to form malonyl-CoA [114]. Malonyl-CoA units are then sequentially added to the growing fatty acid chain by fatty acid synthase (FASN). This process continues through a series of chemical reactions until a long-chain fatty acid is synthesised. The resulting fatty acids can be further modified, incorporated into phospholipids for membrane biogenesis, or stored as triglycerides [115].

Sterol-regulatory element-binding proteins (SREBPs) are transcription factors that play a crucial role in regulating lipid synthesis. SREBP-1 is upregulated along PCa progression [111, 112], partly in an AR-dependent manner [116, 117], and it activates the expression of enzymes involved in de novo lipogenesis, including FASN [118, 119].

Different strategies have been developed to target DNL in PCa. Preclinical studies using SREBP inhibitors such as fatostatin support the idea that targeting this pathway is an interesting strategy to block PCa growth and promote apoptosis [120]. Fatostatin inhibits SREBP cleavage-activating protein (SCAP), a key regulator of lipid metabolism. SCAP is responsible for transporting the SREBPs from the endoplasmic reticulum (ER) to the Golgi apparatus, where they undergo proteolytic cleavage to activate the transcription of genes involved in cholesterol and fatty acid synthesis. By inhibiting SCAP, fatostatin prevents the translocation of SREBPs to the Golgi, thus inhibiting their activation and subsequent transcriptional regulation of lipid synthesis [120]. In addition, dutasteride and C75 are two FASN inhibitors that have been developed and tested for their effectiveness in PCa [121–123]. Dutasteride indirectly reduces FASN mRNA levels by inhibiting the enzyme 5α-reductase, which is responsible for converting testosterone into dihydrotestosterone [121]. C75 inhibits FASN through competitive binding, thus preventing the synthesis of fatty acids from acetyl-CoA and malonyl-CoA [122].

Despite various efforts to target lipid synthesis, a critical challenge persists in elucidating precise biomarkers and methodologies for the identification of lipogenic tumours and the stratification of patients likely to exhibit optimal responses to DNL targeting. For that reason, new targets of the DNL pathway are currently being explored for therapeutic purposes. Recently, a large-scale analysis revealed that the fatty acid elongase ELOVL5 is upregulated in PCa and its depletion leads to antitumoral responses [124]. Concomitantly, the ELOVL5 enzyme also generates polyunsaturated fatty acids (PUFAs), which have been associated with enzalutamide resistance during neuroendocrine differentiation (NED) by activating the AKT-mTOR pathway [125].

#### Lipolysis and fatty acid oxidation (FAO)

Lipolysis refers to the process that converts stored fats or triglycerides into glycerol and fatty acids. In the context of PCa, lipolysis is upregulated to generate fatty acids that are subsequently used as an energy source and building blocks for cellular components [126]. However, lipolysis is a more complex process than lipid synthesis. It requires a balance between fatty acid catabolism, necessary for biomass, and the need for ATP and NADPH production. Elevated levels of monoacylglycerol lipase (MAGL) in AR-independent prostate cancer contribute to malignancy through endocannabinoid and fatty acid pathways [127]. Complementarily, adipose triglyceride lipase (ATGL) expression correlates with worse prognosis in CRPC patients [128]. Inhibition of ATGL impairs PCa cell growth in vitro and in vivo, inducing a metabolic shift towards glycolysis [128].

After being released from storage units, lipids can be catabolised through fatty acid oxidation (FAO), a process where cells utilise FAO as an energy source, and that is altered in PCa [129]. CPT1, the enzyme that transports medium-long fatty acids into the mitochondria for oxidation, is upregulated in PCa [130, 131]. In addition, FAO could sustain a castration-resistant state, which has been demonstrated recently through the inhibition of 2,4-dienoyl-CoA reductase (DECR1) [132, 133].

#### Fatty acid uptake and transport

Fatty acid transport proteins (FATPs) and fatty acid binding proteins (FABPs) are responsible for the uptake of exogenous and intracellular transport of fatty acids, respectively. These proteins are upregulated in PCa, which theoretically increases fatty acid availability for cellular processes [134, 135]. FABP5 inhibition provides a synergistic effect in combination with chemotherapy [136], and the reported dependence of *PTEN* loss-driven PCa [137] on this enzyme encourages the evaluation of this therapeutic strategy in a stratified population. Indeed, SBFI-103, a competitive inhibitor of FABP5, is effective and well-tolerated both in vitro and in vivo in PCa cells resistant to ADT or taxanes [137]. Finally, CD36, a multifunctional cell surface receptor that imports fatty acids, contributes to various aspects of PCa biology, including tumour growth, angiogenesis, and metastasis. The tumour suppressive consequences of *Cd36* deletion in *Pten* loss-induced PCa [138] suggest that knowledge and therapeutic strategies reported for other tumour types could be implemented in this disease [139, 140]. FA6.152, an anti-CD36 neutralising antibody, inhibits all known functions of CD36, including its interactions with thrombospondin, collagens, and fatty acids. Similarly, another CD36 targeting antibody named JC63.1 selectively blocks uptake of fatty acid and oxidised low-density lipoproteins. Treatment of oral squamous cell carcinoma (OSCC) models with these two antibodies impair metastasis [139].

#### Cholesterol metabolism

PCa cells often exhibit increased de novo cholesterol biosynthesis [141–143], and AR signalling controls the expression of cholesterol biosynthetic enzymes, such as HMG-CoA reductase (HMGCR) [144]. The relevance of this pathway in PCa spans multiple biological aspects. First, cholesterol is a critical precursor for the synthesis of steroid hormones, including androgens, which sustains the activation of AR in tumour cells after castration therapy [145–147]. Second, cholesterol is a critical component of lipid rafts, membrane microdomains that play a role in cellular signalling. Alterations in cholesterol levels affect lipid raft dynamics and the associated signalling pathways involved in PCa progression [141]. Third, cholesterol esters are abundant components of lipid droplets, whose presence is associated with PCa aggressiveness [148]. Given the relevance of cholesterol metabolism in cancer, different therapeutic strategies have been proposed for PCa. Statins are cholesterol-lowering agents that are administered chronically to millions of people around the globe. Since they inhibit HMGCR, their potential anticancer activity has been broadly studied [149]. In this regard, high doses of statins in vitro consistently reduce PCa aggressiveness [150, 151]. However, low doses of some of these drugs (equivalent to the concentrations reached in the blood of treated individuals) exhibit paradoxical effects on tumour cells in vitro and in vivo [152]. This discrepancy is evident in epidemiological studies monitoring the influence of statin treatment in PCa pathogenesis and progression [149], suggesting that we still miss critical biological information regarding how these drugs operate in cancer. Cholesterol metabolism could be particularly relevant when targeting androgen production or signalling in PCa. Indeed, inhibition of squalene epoxidase (SQLE), a crucial enzyme in cholesterol biosynthesis, has been proposed as a promising pharmacological intervention for treating CRPC [153, 154]. Targeting SQLE with terbinafine effectively inhibited orthotopic tumours growth in mice. Moreover, in a clinical setting, terbinafine demonstrated the ability to decrease prostate-specific antigen (PSA) levels in three out of four late-stage prostate cancer patients [154]. Similarly, the pharmacologic blockade of SQLE with FR194738 attenuated the growth of PC3 cells both in vitro and in mouse xenograft models [153]. Finally, a complementary strategy to support androgen synthesis in conditions of hormone deprivation is the provision of cholesterol by the TME. In this line, macrophages can serve as a source of cholesterol for PCa cells in the context of androgen deprivation, hence supporting the development of CRPC [155].

### One-carbon metabolism

**Fig. 3:**
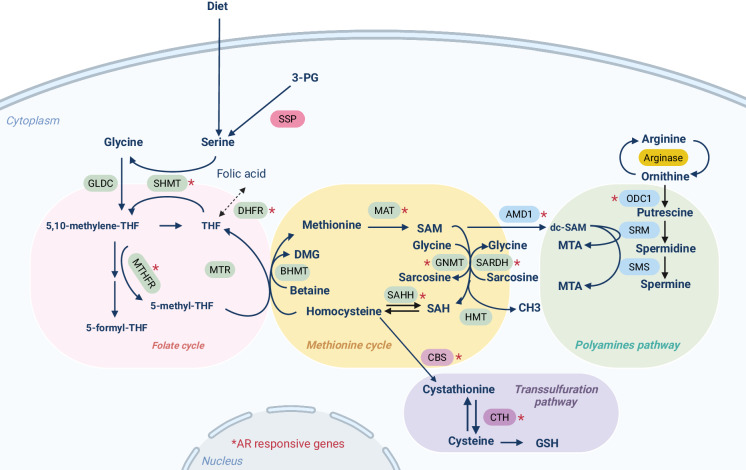
1 C metabolism in PCa. Main metabolites and enzymes involved in 1 C metabolism. This pathway encompasses the folate and methionine cycles, essential for cellular processes like DNA synthesis and methylation. Additionally, it links to the transsulfuration pathway, maintaining redox power through glutathione synthesis, and influencing translation and proliferation via the polyamine biosynthesis pathway. Red asterisks indicate AR-responsive enzymes described in the literature. Abbreviations: 3-PG 3-phosphoglycerate, THF tetrahydrofolate, DMG dimethylglycine, SAM S-Adenosylmethionine, dc-SAM decarboxylated S-Adenosylmethionine, SAH S-adenosylhomocysteine, MTA 5’-methylthioadenosine, GSH glutathione. Created with BioRender.com.

One-carbon (1 C) metabolism involves two central cycles: the folate cycle and the methionine cycle [156]. In the folate cycle, tetrahydrofolate (THF) acts as a carbon carrier for purine and thymidylate synthesis. Methyl groups transfer from 5-methyl THF to homocysteine, forming methionine and connecting the two cycles. Methionine is converted to S-adenosyl-methionine (SAM), a universal methyl donor for protein and DNA methylation. SAM is then metabolized to S-adenosyl-homocysteine (SAH) and later to homocysteine, completing the cycle. Homocysteine produces cystathionine in the transsulfuration pathway, a precursor of glutathione. SAM can also feed into the polyamine biosynthesis pathway through its decarboxylation by S-adenosylmethionine decarboxylase (AMD1) [157] (Fig. 3). Alterations in 1 C metabolic homeostasis are at the core of different diseases including cancer [156]. Tumour cells depend on 1 C metabolism for DNA synthesis, redox balance, methylation reactions and polyamine biosynthesis. All these processes are relevant across different cancers and contribute to tumour progression [158, 159].

In PCa, androgen signalling regulates the activity of 1 C enzymes involved in SAM homeostasis, the transsulfuration pathway and polyamine biosynthesis [160]. In turn, changes in AR activity occurring upon PCa progression and therapy can influence 1 C metabolism and the intricate epigenetic crosstalk [160].

#### SAM homeostasis

GNMT and mitochondrial SARDH are critical enzymes that control SAM availability. They are regulated by androgen signalling and are frequently altered in PCa [161, 162]. GNMT transfers a methyl group from SAM to glycine to form SAH and sarcosine, whereas SARDH demethylates sarcosine to form glycine [163]. These two reactions determine the SAM:SAH ratio for the maintenance of epigenetic responses, and the production of sarcosine in this metabolic step has been proposed as a biomarker in PCa, although this data generated intensive controversy in the field [164, 165]. GNMT, is reported to be both upregulated and downregulated depending on the study, thus suggesting a multifactorial regulation in the different stages of the disease [166, 167]. A feasible explanation relates to regulating GNMT by signalling pathways that exhibit reciprocal negative feedback regulation [168, 169]. AR has a predominant role in sustaining GNMT expression, whereas PI3K activation induces its repression [170], a process that could depend on FOXO regulation, according to studies in *Drosophila melanogaster* [171]. Interestingly, *Gnmt* levels are profoundly reduced in *Pten* loss-driven murine PCa, but a germline deletion of the metabolic enzyme reduced PCa incidence in this model, thus suggesting that either residual GNMT activity is essential for tumorigenesis or that this enzyme plays a critical role in the TME [170]. Finally, a recent study has shown a mTORC1/ATF4-driven downregulation of protein kinase C (PKC)^λ/ι^ in neuroendocrine prostate cancer that increases serine biosynthesis. This metabolic shift supports cell proliferation and elevates intracellular SAM levels, promoting epigenetic changes characteristic of this aggressive form of PCa [172].

#### The transsulfuration pathway

The transsulfuration pathway is a branch of 1 C metabolism that converts homocysteine to cysteine. This process involves several enzymatic steps, with cystathionine beta-synthase (CBS) playing a predominant role [173]. CBS activity is controlled by SAM pools to direct homocysteine towards remethylation when SAM levels are low [173]. In PCa, studies showing both increased and decreased expression of CBS have been published [174, 175]. Lower enzyme levels are found in metastatic PCa cell lines, but these data do not correlate with clinical evidence reporting increased homocysteine and cystathionine abundance in patients with worse outcomes [176, 177]. In this line, cystine depletion sensitises PCa cells to immune checkpoint inhibitors as well as to DNA damage-inducing agents, further highlighting the importance of these intermediates in PCa [178].

#### Polyamine biosynthesis

Polyamines (PA) are small polycations essential for normal cell growth in all eukaryotic organisms [179]. Putrescine is generated from the urea cycle through decarboxylation of ornithine by ornithine decarboxylase (ODC1), whereas AMD1 decarboxylates SAM to dcSAM. This reaction provides the propyl amines necessary to form spermidine and spermine from putrescine through the action of spermidine synthase (SRM) and spermine synthase (SMS) [180]. The prostate epithelium synthesises high levels of polyamines that are secreted into the seminal fluid. Androgens control this process through transcriptional regulation of ODC1 and AMD1 [181, 182]. Accordingly, androgen deprivation therapies reduce the abundance of spermidine and spermine [183]. However, regulation of PA biosynthesis in PCa extends beyond AR signalling. On the one hand, ODC1 is a main target of MYC, which associates MYC amplification and overexpression with elevated polyamine biosynthesis [184]. The regulation of polyamine biosynthesis downstream MYC contributes to the tumour suppressive activity of PGC1α, which was recently reported to repress this oncogene [185–187]. On the other hand, PI3K-mTORC1-dependent regulation of AMD1 stability influences polyamine synthesis [188], an observation that is extensible to other pathophysiological contexts beyond cancer [189].

## Tumor cell-extrinsic metabolic influences

**Fig. 4:**
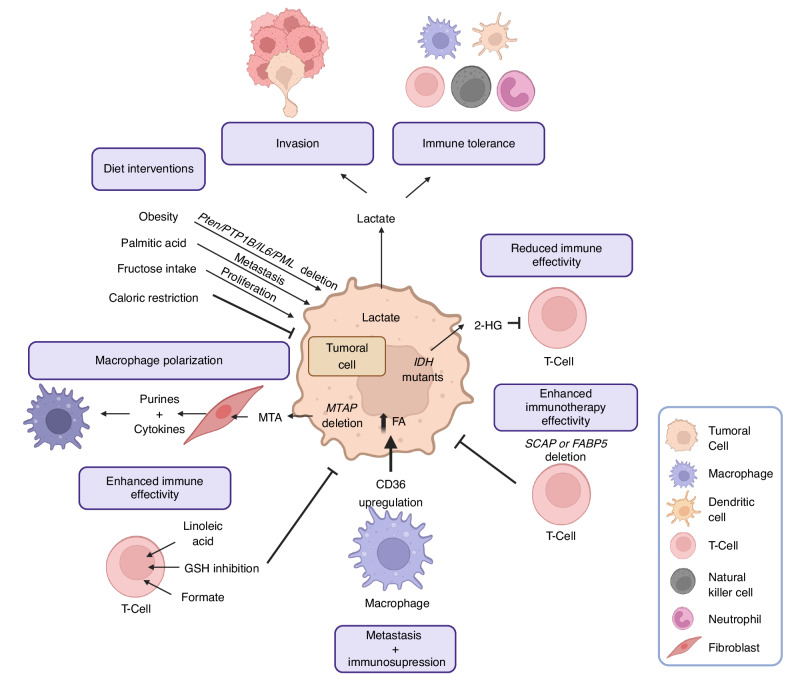
The role of metabolism in the cancer tumour microenvironment. Summary of the recent concepts regarding the interaction of tumour metabolism with the tumour microenvironment to support cancer progression. Abbreviations: 2-HG 2-Hydroxyglutarate, GSH glutathione, IDH isocitrate dehydrogenase, MTAP S-methyl-5’-thioadenosine phosphorylase, MTA 5′-deoxy-5′-methylthioadenosine. Created with BioRender.com.

Prostate cancer is associated with ageing, and in turn, the organism and cellular environment represent an important modifiable factor in the pathogenesis and progression of the disease. There is an emerging interest in studying the metabolic properties of the tumour microenvironment, as well as how exogenous factors like the diet may impact tumour progression. The TME closely interacts with tumour cells and comprises immune cells, fibroblasts, blood vessels, and the extracellular matrix [190]. Immune cells within the TME can either trigger pro-tumoral or anti-tumoral responses [191], while the extracellular matrix and stromal cells within the TME provide structural and biochemical support to tumours, influencing their ability to invade surrounding tissues and metastasize [190]. Advances in high-throughput, single-cell resolution technologies have significantly enhanced our comprehension of cellular diversity in PCa [192–194]. However, there is still very little knowledge about the metabolic adaptations in PCa stromal cells, and a glimpse at other tumour types can provide critical information on what is to come (Fig. 4).

### Glucose metabolism in the TME

The elevated glycolytic rate of tumour cells is directly responsible for creating the acidic and nutrient-depleted conditions of the TME, which have profound consequences for immune activity [195, 196]. One of the most significant effects of aerobic glycolysis is the acidification of the TME due to lactate secretion [197, 198], which supports increased migration and invasion [199] and promotes immune reprogramming towards a tolerant phenotype [196, 200]. Glycolysis in the tumour stroma is also required for adequate antitumoral response, which has led to the development of metabolite-based formulations in the presence of a glycolytic inhibitor that specifically targets cancer cells [201]. Cancer and T cells compete for glucose among several other metabolites, and the avidity of cancer cells for this nutrient diminishes the cytolytic activity [195, 202, 203]. Glycolytic capacity in T cells is also influenced by oncometabolites such as 2-hydroxyglutarate, which is produced at high concentrations in isocitrate dehydrogenase mutant cancers and inhibits their proliferation, cytokine production, and ability to kill tumour cells [204].

### Lipid metabolism in the TME

Lipid metabolism is similarly required in stromal cells. SREBP activity orchestrates the immune responses in cancer. Inhibition of SREBP function in regulatory T cells (Treg) enhances antitumour immune responses [205]. Particularly, SREBP-cleavage-activating protein deletion in intra-tumoral Tregs inhibits tumour growth and improves PD-1-triggered immunotherapy by regulating interferon-γ production [205]. Similarly, deletion of *FABP5* in Treg affects mitochondrial integrity and triggers cGAS-STING-dependent type I IFN signalling [206]. Macrophages are regulated by tumour cells at multiple levels and their polarisation contributes to the acquisition of aggressive features. Upregulation of *CD36* in metastasis-associated macrophages (MAMs) promotes tumour cell-derived fatty acid uptake, protumoural polarization and their supportive role in the establishment of liver metastasis [207]. Thus, targeting CD36 emerges as a two-hit strategy targeting both tumour and immune cells in the treatment of metastasis. Lipids can also support the activation of lymphocytes. As an illustrative example, linoleic acid activates CD8 + T cells, enhancing metabolic fitness and preventing exhaustion [208], highlighting its role as a potential adjuvant to potentiate adoptive T cell therapy.

### One-carbon metabolism in the TME

Very little is known about the contribution of 1 C metabolism to the TME in PCa. In turn, scattered evidence in other tumour types can provide an idea of the processes influenced by this metabolic route in cancer. Deficiencies in one-carbon metabolism impair the effectiveness of PD-1 blockade in melanoma. Coherently, augmenting 1 C metabolism through formate supplementation during anti-PD-1 therapy improves CD8 + T-cell fitness and facilitates CD8 + T-cell-mediated tumour clearance [209]. These results indicate that formate supplementation has the potential to enhance the function of exhausted CD8 + T cells. Importantly, the acidification of the extracellular milieu also influences T cell function, eliciting a reduction in methionine metabolism via SLC7A5 downregulation that results in a ‘stem-like memory’ state. This reprogramming enhances T cell persistence and anti-tumour efficacy in mice, revealing a novel influence of acidic conditions on T cell characteristics [210]. The relevance of 1 C metabolism for glutathione production and redox balance is an additional factor controlling the activity of the TME. Disrupting glutathione synthesis in Tregs impairs their ability to regulate serine metabolism, leading to severe autoimmunity and improved anti-tumour responses [211]. Tumour-intrinsic 1 C metabolism produces secreted metabolic intermediates that can remodel the TME. Tumour cells exhibit frequent loss of methylthioadenosine phosphorylase (MTAP), which leads to the accumulation of its substrate MTA [212]. Secreted MTA is uptaken and metabolized by fibroblasts, which will produce and secrete both purine products and cytokines that induce macrophage polarization.

### Diet and obesity

Nutrition represents the tightest interaction of our organism with the environment. As such, it is closely linked to the development of diseases, including cancer. Studies in other tumour types have unveiled additional molecular processes responsible for the high-fat diet-induced phenotype. In oral carcinoma and melanoma models dietary palmitic acid, but not oleic or linoleic acid, promotes metastasis in mice [139, 140]. Molecularly, palmitic acid induces a pro-metastatic memory involving CD36, histone modifications, and a neural signature linked to Schwann cells, leading to both metastasis initiation and long-term metastatic memory. These same modifications may also play a role in PCa [140]. In line with the role of CD36, a high-fat diet has been shown to promote metastasis by enhancing saturated fatty acid uptake via this receptor in breast cancer [213]. Modifications in dietary habits could also be beneficial for cancer patients. Caloric restriction induces anti-proliferative effects in mouse xenografts, an effect that is limited to tumours without mutations causing constitutive activation of the PI3K pathway [214]. More recently, caloric restriction has been shown to inhibit the growth of certain tumours in mice by lowering lipid levels in both plasma and tumours [215]. This dietary modification reduces stearoyl-CoA desaturase activity in cancer cells, causing an imbalance between unsaturated and saturated fatty acids and impairing tumour growth.

In PCa obesity has been linked to an increased risk and progression of the disease in epidemiological studies [216–218], owing to the contribution of factors such as insulin resistance, chronic inflammation, or hormonal dysregulation, among others. However, the causal contribution of obesity to PCa and the mechanistic foundations of this effect remains elusive. Murine models have shed some light on these questions. Obesity and high calorie-induced hyperinsulinemia promote PCa in prostate-specific *Pten*^-/-^ mice by increasing cell proliferation and activating insulin/IGF1/PI3K/AKT signalling pathways [219, 220]. In line with this notion, mutations in PCa that activate PI3K (such as prostate-specific *Pten* loss) prime or promote obesity-driven PCa aggressiveness in conjunction with other signalling pathways, such as loss of *Ptpn1* [221], IL6/pSTAT3 signalling activation [222] or *Pml* co-deletion [223]. This knowledge offers new therapeutic opportunities for targeting PTP1B, IL6 or PML-loss induced SREBP signalling in the context of obesity.

Although much of the emphasis on the influence of obesity has been put on lipid availability and chronic inflammation, sugars could also play a relevant role. Indeed, increased expression of fructose transporters in PCa has been suggested to promote fructose uptake and metabolism to support cancer cell fitness [224].

Collectively, dietary interventions may also play a role in both the progression and treatment of PCa, and further studies are required to extend the knowledge of molecular and biological effectors that can be translated into preventive and therapeutic actions.

## Concluding remarks and open questions

Over the past decade, there have been extensive efforts to understand the mechanisms and biological consequences of metabolic reprogramming in cancer. Although currently there are no drugs approved for PCa treatment that target specific metabolic pathways, there are multiple agents in development. Metabolic reprogramming is essential for the biology of cancer cells. Tumour metabolism is influenced by cancer cell-specific metabolic adaptations as well as by metabolic alterations in the TME. Modern technologies to study metabolism, including new imaging techniques, spatial metabolomics and single-cell RNA sequencing have redefined our knowledge of cancer metabolism. However, despite extensive research in PCa metabolism, there is still a gap in knowledge on the therapeutically-actionable metabolic pathways that are relevant to each stage of the disease. Further research into the metabolic dependencies of the primary tumour and those of the metastatic lesions, including the role of ferroptosis, hypoxia and microbiota, might lead to new metabolic interventions to prevent metastatic dissemination of prostate cancer, and to significant improvements in the curation rate of this disease.
